# Colonization and extinction mediate environmental effects on the phylogenetic diversity of invertebrate communities

**DOI:** 10.1002/ecy.70400

**Published:** 2026-05-08

**Authors:** Nadia B. Páez‐Rosales, Jessica L. Ware, Diane S. Srivastava

**Affiliations:** ^1^ Department of Zoology and Biodiversity Research Centre University of British Columbia Vancouver British Columbia Canada; ^2^ Division of Invertebrate Zoology American Museum of Natural History New York New York USA

**Keywords:** bromeliad ecosystem, community phylogenetics, experimental ecology, habitat size, predation, top‐down trophic interactions, tropical ecology

## Abstract

Phylogeny offers a powerful framework for understanding mechanisms driving community assembly. Yet, most empirical studies in community phylogenetics rely on observational approaches. In this study, we explore how two important drivers of community assembly—habitat size and predator presence—shape species richness and phylogenetic relatedness of prey communities by altering colonization and extinction processes. Using bromeliad invertebrate communities as our study system, we combined surveys of natural communities with experiments that manipulated habitat size and predator presence. Colonization and extinction were isolated in separate experiments to test whether effects of habitat size and predator presence differed across stages of community assembly. Following species–area theory, we expected larger habitats to increase species richness and, given the strong consumptive effects of the top predator (a damselfly larvae), we expected species richness to decline in the presence of predators. Under a community phylogenetics framework, if traits mediating responses to these factors are phylogenetically conserved, we expected the phylogenetic structure of the community (i.e., relatedness) to have deterministic patterns along both gradients. Specifically, if habitat size functions as an environmental filter, small bromeliads would host phylogenetically clustered assemblages; alternatively, if it functions as a mediator for coexistence among close relatives, larger habitats would exhibit greater relatedness. Likewise, we expected the generalist top predators to increase relatedness when closely related taxa have shared defensive traits. As traits mediating community assembly may vary in their phylogenetic distribution across lineages, we also anticipated relatedness patterns to vary across taxonomic scales. We found a positive effect of habitat size on species richness, which was driven by colonization mechanisms. Habitat size also affected relatedness, but the direction depended on the taxonomic scale, with positive relationships at broad scales and negative relationships at narrower scales. By contrast, predators reduced species richness through extinction mechanisms, although these effects were masked in natural communities by continuous replacement of individuals through colonization. Predator effects on relatedness were variable across taxonomic scales, suggesting the involvement of multiple traits at different phylogenetic depths. Together, our findings highlight the complex interplay between environmental factors and community assembly in structuring taxonomic and phylogenetic dimensions of diversity.

## INTRODUCTION

One of the core goals of community ecology is understanding how the abiotic and biotic environment regulates community diversity. Most research has focused on taxonomic metrics (e.g., species richness); however, these overlook species' evolutionary and functional differences, implicitly assuming them as equivalent or interchangeable (Ives & Helmus, 2010; Pavoine & Ricotta, 2014). In this study, we additionally consider the evolutionary similarity of species in the community, that is, relatedness, which can provide deeper insights into assembly mechanisms (Kraft et al., 2007; Webb et al., 2002). For example, when phylogenetic relatedness covaries with environmental conditions, it suggests that ecological processes shape the evolutionary similarity among co‐occurring species (Cavender‐Bares et al., 2009; Webb et al., 2002). Likewise, when traits are phylogenetically conserved—that is, related species are more similar in their traits than distantly related species—relatedness can be informative of functional similarity in the community (Tucker et al., 2018). Here, we evaluate, both observationally and experimentally, how an abiotic (habitat size) and a biotic factor (predator presence) influence the species richness and relatedness of a community.

Environmental drivers can influence communities by altering rates of colonization—here defined as the arrival of new species into a community—and extinction—the local loss of species. Colonization and extinction dynamics have long been recognized as central to theories of community assembly, including island biogeography (MacArthur & Wilson, 1967), patch dynamics (Levin & Paine, 1974), and metacommunity theory (Leibold et al., 2004). Disentangling their effects is essential for a mechanistic understanding of community assembly. While observational research enables the assessment of diversity patterns and their association with environmental factors under natural conditions, it is difficult to infer mechanisms from observational data alone, as multiple ecological processes can act simultaneously. Therefore, we use experiments to assess the effects of key environmental variables under controlled conditions, isolating their influence on colonization and extinction separately and then relating these processes back to patterns observed in nature. Such coupled observation–experimental approaches remain rare in community phylogenetics (Cadotte & Tucker, 2017; Kraft et al., 2015), especially in animal communities.

**FIGURE 1:**
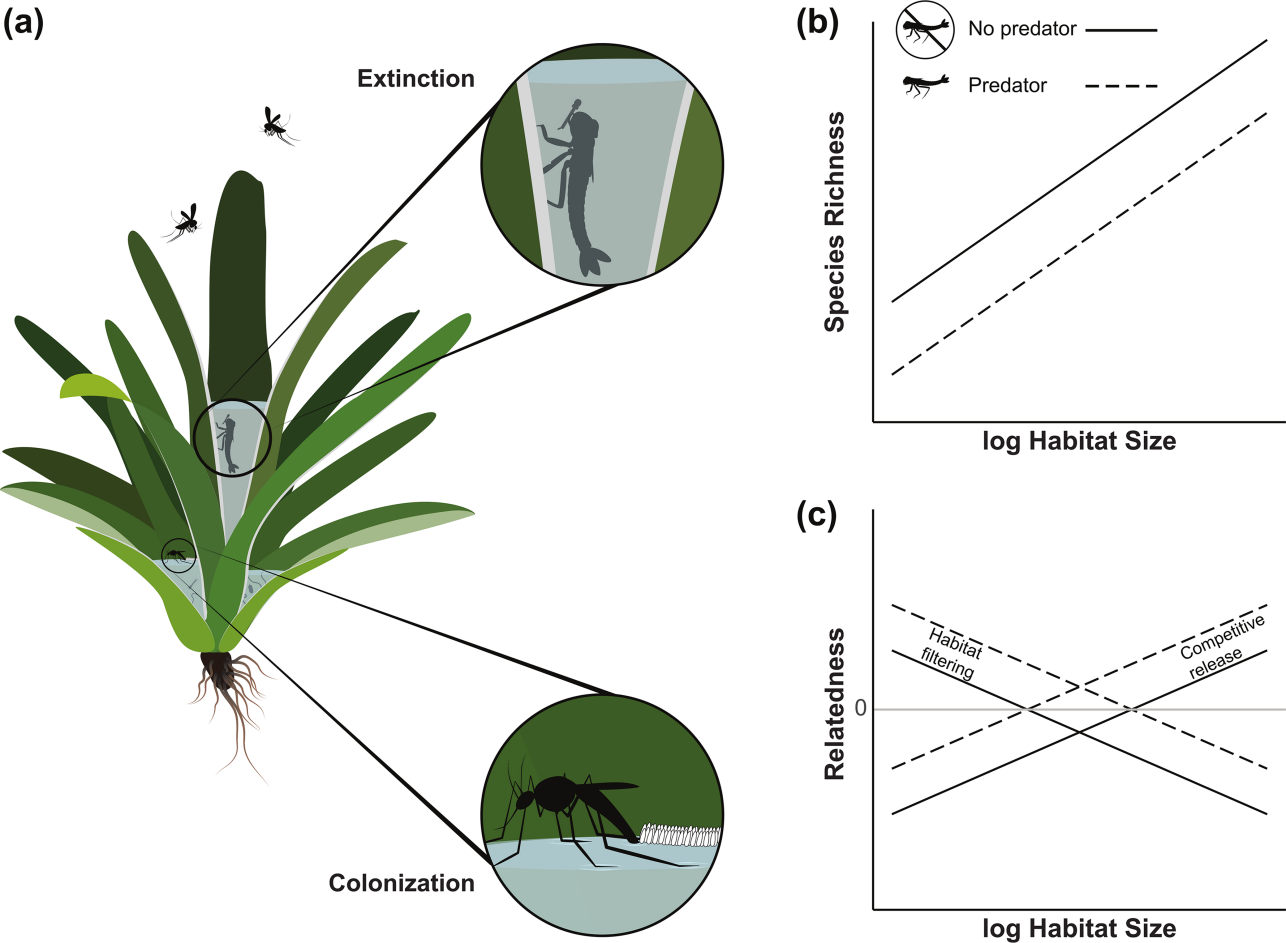
Hypothesized effects of habitat size and predator presence on the community's species richness and relatedness. (a) Illustration of the colonization and extinction processes in the aquatic ecosystem of bromeliads. The top circle illustrates the consumptive effects of damselfly larvae, increasing the risk of local extinction. The circle on the bottom shows colonization through oviposition of a flying adult. (b and c) Diagrams with hypothesized effects of habitat size and predator presence, respectively. Lines represent predicted relationships between habitat size and species richness or relatedness in habitats with (dashed) and without (solid lines) predators. Illustration by Sylvia Heredia.

Habitat size is one of the best‐documented determinants of species richness, with most explanations for species–area relationships involving processes that increase colonization or reduce extinction in large habitats. For example, larger habitats may be more readily detected by potential colonizers or passively intercept more propagules (McCormick et al., 1988). Large habitats can also contain more resources, which support larger effective population sizes, thereby reducing extinction risk from stochastic events. Finally, large habitats can also include more resource/niche heterogeneity, facilitating the coexistence of multiple species (MacArthur & MacArthur, 1961; Root, 1973; Williams, 1964). Beyond effects on species richness, habitat size can also influence phylogenetic structure (Morlon et al., 2011). We consider two alternative hypotheses for the effects of habitat size on relatedness (Figure 1). On the one hand, if small habitats act as environmental filters—for example, if limited resources or stressful environmental conditions exclude species lacking particular conserved traits—relatedness should decrease with habitat size (Matthews et al., 2020). On the other hand, larger areas may weaken biotic interactions such as competitive exclusion by encompassing greater resource heterogeneity and spatial complexity, thereby facilitating the coexistence of close relatives and leading to a positive relationship between habitat size and relatedness (Cavender‐Bares et al., 2009; Helmus & Ives, 2012).

Trophic interactions can also impact community diversity, especially at smaller spatial scales (McGill, 2010). Research in community phylogenetics has been conducted mainly on large spatial scales, where the effects of other trophic levels are more difficult to observe and are therefore less explored. Here, we focus on the effect that predators have on prey communities at local scales. By reducing the abundance of dominant competitor species, predators can increase coexistence (Caswell, 1978). Conversely, if such reductions in abundance exceed the ability of recruitment or immigration to sustain populations, predation will decrease species richness. Predators can also affect community composition through non‐consumptive effects; for example, their presence can alter prey behavior by deterring colonization, inducing emigration, or changing activity patterns (Blaustein, 1999; Werner & Peacor, 2003). It has been proposed that generalist predators can lead to phylogenetic clustering (co‐occurrence of closely related species) when prey defense mechanisms are conserved, or to phylogenetic overdispersion (co‐occurrence of distantly related species) when defense responses are convergent across lineages (Cavender‐Bares et al., 2009; Fine et al., 2004). When predator preferences are more specialized or multiple antipredator strategies are equally good, prey communities may have more phylogenetically dispersed or random structures (Cavender‐Bares et al., 2009). The effects of predators can therefore lead to different patterns in prey relatedness, depending on the interaction between predator specialization and the phylogenetic distribution of prey defenses.

To understand how habitat size and predator presence influence phylogenetic diversity, we need to experimentally manipulate each factor and isolate their effects on colonization and extinction processes. An ideal system for this is the aquatic invertebrate community within bromeliads. These aquatic microcosms host a diverse macroinvertebrate community that forms complex trophic networks, offering sufficient taxonomic variation for phylogenetic analyses and allowing experimental manipulation of trophic interactions. Their small size enables complete community sampling and high replication and facilitates the manipulation of environmental factors and the isolation of stages of community assembly (Srivastava et al., 2004).

In the bromeliad system, both habitat size and predator presence have been shown to strongly affect species diversity (Dézerald et al., 2014; Rezende et al., 2020). The positive trend between species richness and habitat size, analogous to the species–area relationship, has been mainly explained by species' physiological limitations: Small bromeliads are prone to drought, and species with low drought tolerance are excluded from the community either through direct mortality or by avoiding colonization of small bromeliads (Amundrud & Srivastava, 2015). Beyond effects on species richness, habitat size can also influence community relatedness through distinct mechanisms. Smaller bromeliads may act as habitat filters, restricting colonization and survival to species with specific adaptations, such as drought tolerance. If these traits are phylogenetically conserved, relatedness would decrease with bromeliad size (Webb et al., 2002). Alternatively, bromeliad size may influence relatedness by regulating competition: If larger bromeliads facilitate the coexistence of closely related species by reducing competition, community relatedness would increase with bromeliad size.

Like habitat size, top‐down control is a key driver of local diversity, with odonate larvae representing the top predators of the bromeliad system (Petermann et al., 2015). These generalist predators are capable of consuming almost every species within the community (Srivastava, Ware, et al., 2020), reducing prey abundance and leading to decreased prey richness (Amundrud & Srivastava, 2016). Defense mechanisms against odonate larvae can occur at different life stages in the prey, influencing distinct assembly processes. Adults may refrain from ovipositing in predator‐containing habitats if they are able to detect chemical cues of damselfly larvae in the water (Hammill, Atwood, & Srivastava, 2015), whereas larvae may use behavioral and physiological responses to avoid detection, thereby reducing extinction risk (Hammill, Atwood, Corvalan, & Srivastava, 2015; Hammill & Beckerman, 2010). These adaptations, however, have been analyzed only for a few prey species in the system, and therefore the phylogenetic distribution of these traits remains unknown. If these defense traits are phylogenetically conserved, predator presence should lead to communities composed of more closely related species.

More generally, community assembly mechanisms may differ throughout the phylogenetic tree, and phylogenetic metrics are often sensitive to the taxonomic scale of analyses (Graham et al., 2018; Swenson et al., 2006). In a diverse community such as ours, analyzing only the entire assemblage may confound interpretations by overrepresenting the dynamics of highly divergent taxa (if their response differs from the rest of the community) or by masking dominant, deterministic patterns driven by specific clades. To account for potential scale dependence in phylogenetic structure (i.e., relatedness), we examine patterns within relevant taxonomic subsets of the community.

In summary, we combine experiments and surveys to examine how habitat size and predator presence affect community species richness and relatedness, testing potential community assembly mechanisms by separating colonization and extinction processes. Based on ecological theory and existing knowledge of our study system, we expect species richness to increase with habitat size and decrease with predator presence. We consider two alternative hypotheses for the effects of habitat size on relatedness: If traits mediating responses to habitat size are phylogenetically conserved and habitat size acts as an environmental filter, we expect prey relatedness to decrease with greater habitat sizes; alternatively, if habitat size mainly acts as a mediator for competitive interactions, relatedness should increase with size. Finally, assuming prey defenses against the top generalist predator are conserved, we expect predator presence to lead to more closely related communities (Figure 1). To help interpret phylogenetic patterns, we assess how species responses to experimental conditions of habitat size and predator presence are distributed across the phylogeny. We additionally test the sensitivity of patterns to the taxonomic scale by analyzing relevant subsets of the community.

## MATERIALS AND METHODS

### Study site and experimental design

We conducted a field survey and two experiments in a premontane rainforest at Pitilla Biological Station, Guanacaste Conservation Area, Costa Rica (600 m above sea level), during the fall of 2019 (September 29–December 9). The survey assessed natural diversity patterns of bromeliad invertebrate communities along habitat size and predator biomass gradients, and the experiments tested the contributions of colonization and extinction processes to these patterns.

In the survey, we sampled 20 bromeliads within a 750‐m radius of the station, each at least 100 m apart, belonging to the genera *Werauhia* J.R. Grant and *Guzmania* Ruiz & Pav. Previous studies with these two genera have shown that bromeliad identity does not explain differences in invertebrate community composition once bromeliad size is accounted for (Ngai et al., 2008). We recorded the water‐holding capacity of each tank (22–1446 mL), which, on a logarithmic scale, is a standard measure of habitat size in this system (Guzman et al., 2021; Srivastava, 2006). To evaluate predator effects, we considered only the top predator at the field site: larvae of the damselfly *Mecistogaster modesta*, since their strong top‐down effects overshadow those of mesopredators (e.g., Atwood et al., 2014; Srivastava, 2006; Starzomski et al., 2010). Given that habitat size and predator presence covary in natural bromeliad communities—damselfly larvae are absent in small bromeliads and increase in abundance with size (Srivastava et al., 2008)—our experiments were formulated to separate the effects of both variables.

In the experiments, we crossed 10 levels of bromeliad size by two predator levels: *M. modesta* larvae present or absent. Bromeliads chosen for experiments belonged to the same species of the genus *Werauhia*. In preparation for the experiments, we collected, emptied, and air‐dried bromeliads for 7 days to completely remove organisms (Appendix S1: Figure S1). The detritus from these bromeliads was pooled, dried at 65°C, and then replaced in experimental bromeliads according to log–log regressions of detritus mass against bromeliad capacity for *Werauhia* bromeliads (*n* = 37). Bromeliads were then filled with unchlorinated bottled water. In the predator‐present treatment, to maintain constant predator pressure in bromeliads of differing size, damselfly larvae were added in numbers predicted for *Werauhia* bromeliads. These predictions originated from a log–log regression relating damselfly abundance to bromeliad size, fitted from previous censuses at the same field site (data from Srivastava, Ware, et al., 2020). Mean predator body size per bromeliad was ≈11.5 mm. We added no *M. modesta* larvae to the predator‐absent bromeliads. Detritus was allowed to rehydrate within bromeliads for 24 h before adding invertebrates; plants were placed in the forest interior close to the station and netted during this period to prevent colonization. Assignment of predator treatment within each bromeliad size level, as well as the position of bromeliads along the transects, was randomized.

For the colonization experiment, we suspended 20 bromeliads (45–1050 mL capacity) in a homogeneous area of rainforest, at least 5 m apart to avoid spatial autocorrelation (see Jocque & Field, 2014). When present, damselfly larvae were individually caged in 50‐mL centrifuge tubes with mesh windows, enabling the chemical cues released by predators to disperse in the water while preventing the consumption of colonizing larvae. Empty cages were used in predator‐absent treatments as controls. Damselfly larvae were hand‐fed every other day with dipteran larvae, and to control for potential colonization effects of researcher presence, we spent equal time near bromeliads without predators. We censused invertebrate communities after 30 days, a time frame chosen to minimize prey loss due to emergence (Hammill, Atwood, & Srivastava, 2015) and to limit the development of strong competitive interactions or consumptive predation that could introduce confounding extinction effects.

In the extinction experiment, we suspended 20 bromeliads (40–490 mL capacity), each containing an initial and standardized prey invertebrate community. Each bromeliad was enclosed in a net (1‐mm mesh; Appendix S1: Figure S1) to prevent further colonization. We assessed survival after 21 days, a duration long enough to detect consumptive effects while avoiding complete depletion of prey communities. Initial prey communities had similar species composition and relative abundances (based on availability), but total prey biomass was scaled to the size of the bromeliad. Specifically, we predicted total prey biomass for the experimental habitat sizes using a log–log regression of prey biomass against water capacity, based on previous survey data for *Werahuia* bromeliads at the field site (*n* = 65). Initial communities contained the 10 most common prey morphospecies in the field site: Naididae sp., *Scirtes* spp., *Trentepohlia* sp., *Polypedilum* spp., *Orthocladiinae* spp., *Anopheles* sp., *Culex* sp., *Wyeomyia* sp., *Wyeomyia* spp. A., *Copestylum* sp. Every invertebrate was introduced in a random compartment of the bromeliad. The bromeliad size range was smaller than that of the colonization experiment due to the difficulty in obtaining sufficient invertebrates for larger bromeliads.

### Trait analyses

To interpret patterns of community phylogenetic structure, we assessed whether species responses to habitat size and predator presence were phylogenetically conserved. These responses were used as proxies for underlying functional traits, with species‐level trait values estimated by quantifying responses to experimental treatments and comparing them to null expectations. Specifically, we evaluated habitat preference and predator avoidance using data from the colonization experiment, and larval sensitivity to habitat size and predator presence using data from the extinction experiment. For each response, we calculated *z*‐scores summarizing deviations of observed species distributions from expectations under a null assembly model. We then mapped species response indices onto the phylogeny and tested for phylogenetic signal. To assess phylogenetic signal and calculate community phylogenetic metrics, we used a purpose‐built phylogeny of focal invertebrates as synthesis phylogenies are poorly resolved for tropical invertebrate taxa (Li et al., 2019). This phylogeny was also required due to unresolved taxonomy in the system, as many bromeliad invertebrates can only be confidently identified with morphology to the family or infraorder level (Céréghino et al., 2018), and cryptic diversity in the system (the prevalence of which was not yet known) could potentially bias diversity metrics. Appendix S1 (Section S1, Table S1) includes a detailed explanation of trait estimation, molecular protocols, and phylogenetic analyses.

### Community analyses

For both the experiments and surveys, we summarized prey communities by species richness, and two relatedness metrics that measure phylogenetic divergence at different depths in the phylogeny: the net relatedness index (NRI), which reflects the overall phylogenetic clustering across the community, and the nearest taxon index (NTI), which measures the proximity among close relatives (Tucker et al., 2017; Webb et al., 2002). Both relatedness metrics were standardized relative to expectations under a null model. We used the *taxa.labels* null model, which shuffles species labels in the bromeliad × species matrix while preserving local species richness. The species pool for the surveys was the total species found in the field site, for local colonizing communities was the total colonizing subset, and for the surviving communities, the species that were manually assembled for the experiment. Diversity metrics were estimated with the *picante* R package (Kembel et al., 2010). Total phylogenetic diversity (Faith's PD)—a function of species richness and phylogenetic dispersion—was highly correlated with species richness (*r* = 0.96), so it was not included for analyses. Since cryptic diversity was determined after the fieldwork, we randomized species identities within morphospecies for non‐sequenced individuals. We assessed the sensitivity of results to abundance by calculating metrics both weighted and unweighted by abundance—we used squared abundances for natural and colonizing communities to better reflect the number of colonization events and to prevent species with large clutch sizes from overshadowing the contributions of other species; in the extinction experiment, we used untransformed abundances instead, as extinction events occur at an individual level.

For our analyses, we fit linear models, using each diversity metric as the response variable of predator biomass, habitat size, and their interaction. Response metrics were standardized so that coefficients could be compared across models. Model assumptions were evaluated using standard diagnostic plots and formal tests. When deviations from assumptions were detected (e.g., non‐normality, heteroscedasticity, nonlinearity), we applied Box–Cox or Yeo–Johnson transformations to the response variable. In the experiments, we ensured that predator and detrital biomass scaled perfectly with habitat size, but this scaling was variable in natural bromeliads. We therefore included predator biomass and detrital biomass as covariates in survey models only. We included linear and quadratic terms for habitat size, that is, the logarithm of bromeliad water capacity. For each response variable, we fit full and reduced models and selected the model with the lowest corrected Akaike information criterion (AICc). In the case of multiple models within 2Δ from the lowest AICc, we chose the model with the fewest variables. Adding detritus as a covariate to our survey models did not substantially modify our interpretation, although in a few cases, it explained additional variation. We excluded from the survey dataset three bromeliads from a different microhabitat, as their invertebrate composition was substantially different, as well as any accidental terrestrial species, and large predators other than damselflies. These predators were not included in the experiments because they are less common and potential prey to damselflies.

Finally, as phylogenetic patterns can depend on the taxonomic scale (i.e., the highest taxonomic level included in the analysis; Swenson et al., 2006), we evaluated how sensitive our results were to analyses at relevant taxonomic subsets of the community. Namely, in addition to the invertebrate level, we focused on the insect (i.e., excluding annelids) and dipteran (i.e., additionally excluding coleopterans) communities, as phylogenetic metrics can be disproportionately influenced by distantly related taxa with long branches, which may obscure phylogenetic patterns. Consistency in trends across taxonomic scales, or between experiments and surveys, would increase confidence in the robustness of our results, even though the power of any individual analysis may be constrained by the sample size (*n* = 17–20). Thus, tests at restricted taxonomic scales should be viewed as checks of robustness of the invertebrate scale models, rather than multiple tests of different hypotheses.

## RESULTS

### Species pool and local diversity

We present one of the first studies to use molecular methods to assess diversity and reconstruct evolutionary relationships for entire bromeliad invertebrate communities (Appendix S1: Figures S2 and S3). We detected 124 species at this site based on molecular species delimitation analysis, a substantial increase from the 57 species previously reported using morphology alone (Srivastava et al., 2023). Natural communities, including all invertebrates, exhibited a near random overall phylogenetic structure (NRI ranging from −0.84 to 2.31, median: −0.2), while close relatives tended to be more related than expected from null models (NTI ranging from −0.33 to 4.18, median: 1.99). Compared to natural communities, invertebrate communities were more clustered in the colonization experiment and more dispersed in the extinction experiment (Appendix S1: Figure S4).

### Habitat size

**FIGURE 2:**
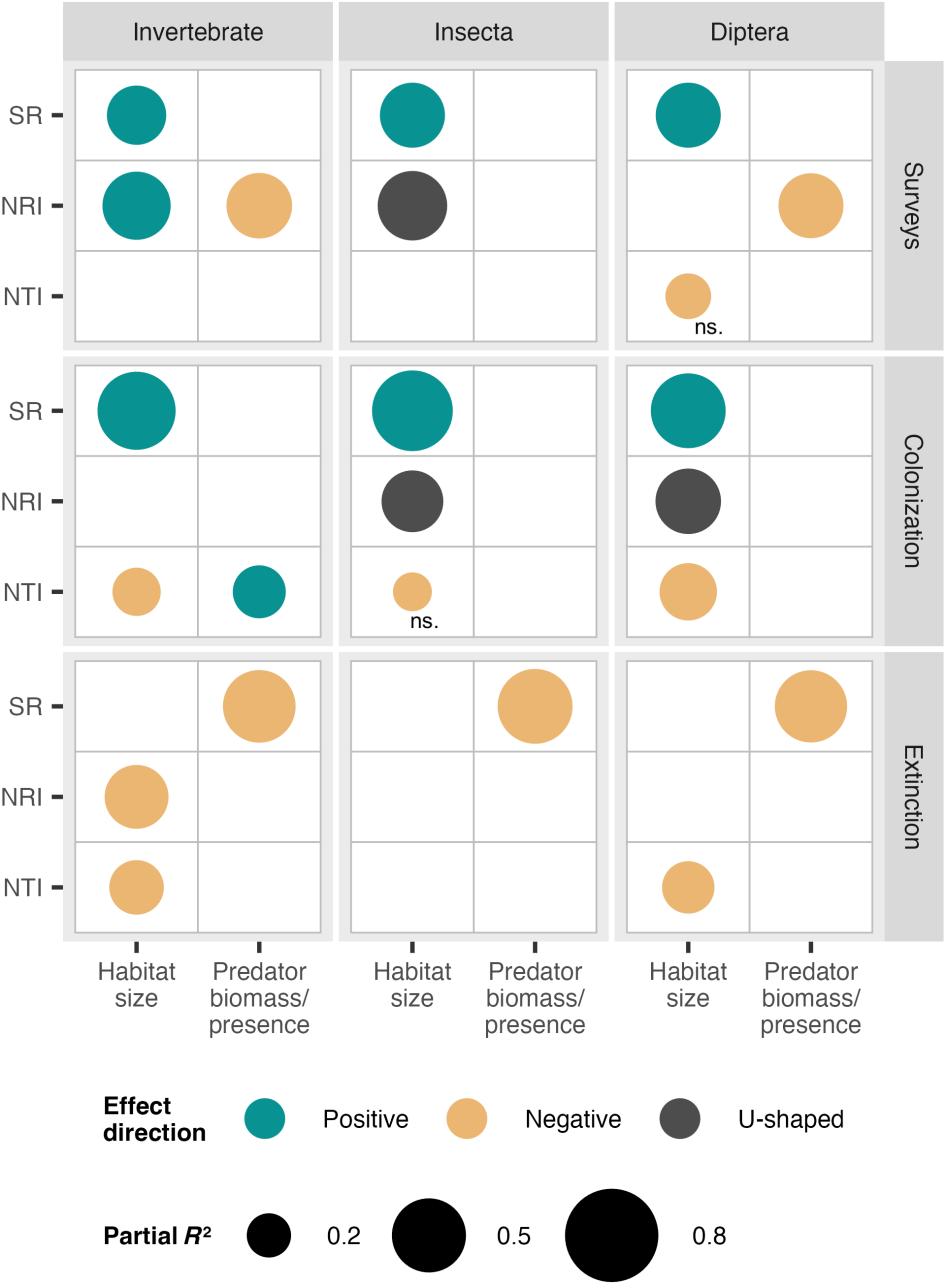
Heatmap with contributions of habitat size and predator variables to the variation of diversity metrics across taxonomic scales: (a) natural communities; (b) experimental colonizing communities; (c) experimental surviving communities. Circle size represents partial *R* ^2^, and color is the direction of the effect. SR is for species richness, NRI for net relatedness index, and NTI for nearest taxon index. When an intersection is empty, the predictor variable is not included in the best model; “ns” indicates the effect is nonsignificant. For complete information on the models, see Appendix S1: Table S2.

**FIGURE 3:**
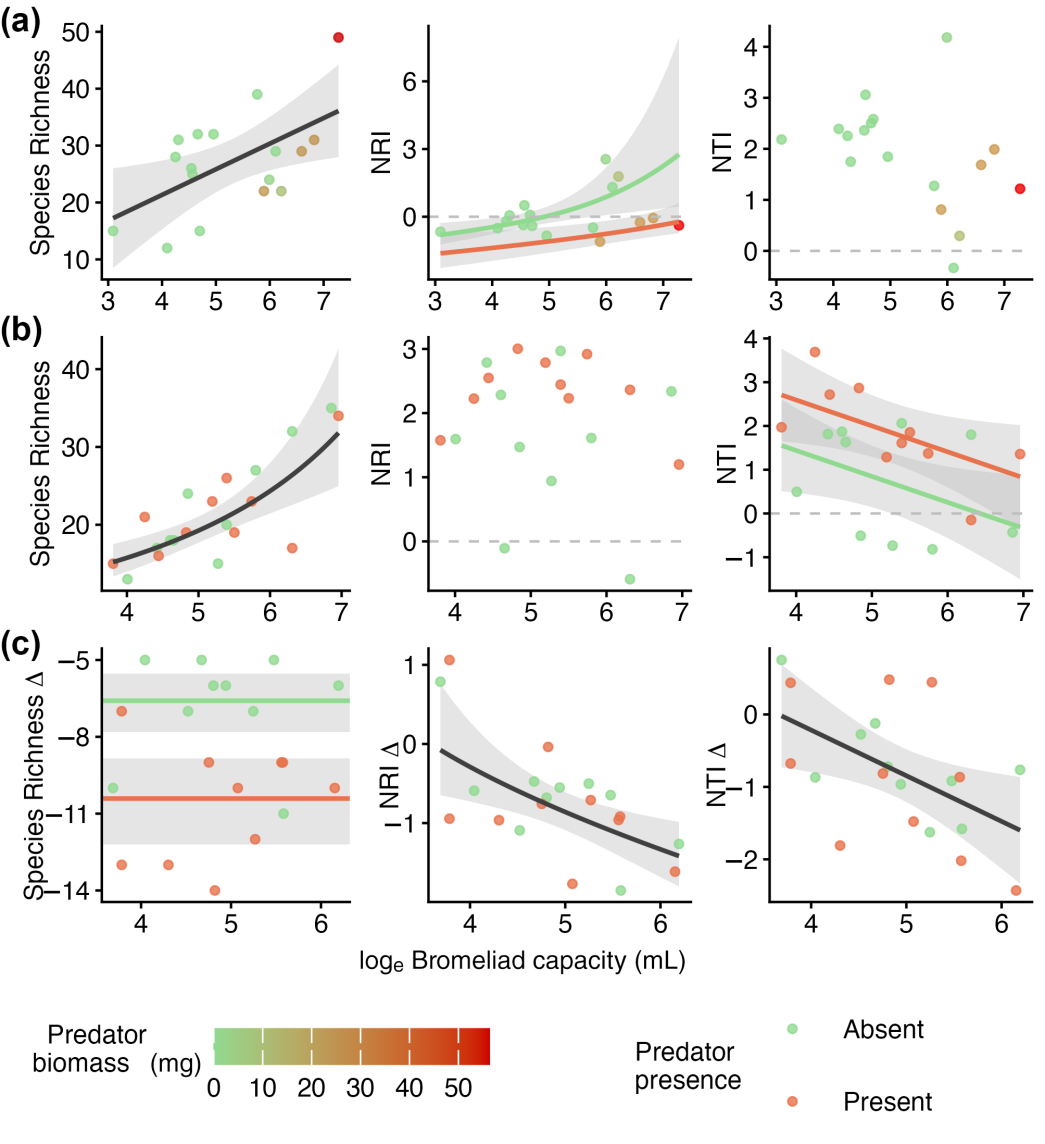
Effects of habitat size and predator biomass on invertebrate diversity metrics. Scatterplots showing species richness and relatedness indices against bromeliad size for (a) natural, and experimental (b) colonizing and (c) surviving communities. Each point represents a local invertebrate community (bromeliad). In (a), points are colored according to the biomass of predators found during surveys. In (b) and (c), points are colored according to predator treatment. Lines represent the model with the best fit for a particular variable, and gray bands represent 95% CIs. NRI = net relatedness index; NTI = nearest taxon index. When the predator variable is included in the best model, lines are colored according to predator levels. If lines are absent, the null model is the best model. Information on model coefficients at this and narrower taxonomic scales is available in Appendix S1: Table S2.

The most ubiquitous pattern we found in naturally assembled communities was an increase in species richness with habitat size, regardless of taxonomic scale (Figure 2, Figure 3; Appendix S1: Table S2). Considering all invertebrates, overall relatedness (NRI) also increased with habitat size—that is, larger tanks have, on average, more closely related species than smaller tanks. However, this pattern changed with taxonomic scale: the Insecta subset had greater relatedness at intermediate habitat sizes, whereas relatedness of the Diptera subset did not depend on habitat size. When considering the relatedness among close relatives in the community (NTI), habitat size had no effect on the Invertebrate and Insecta scales, but had a marginally negative effect on close‐relative relatedness among Diptera—that is, neighbor dipteran species were more closely related in smaller bromeliad tanks (Figure 2; Appendix S1: Table S2).

We then related these patterns in naturally assembled bromeliads to experimentally isolated colonization and extinction processes. Regardless of taxonomic scale, larger habitat size facilitated the colonization of more species—as observed in the colonization experiment—matching patterns in natural communities; however, habitat size did not affect the number of surviving species in the extinction experiment (Figure 2). The effects of habitat size on relatedness were variable and often did not match those of natural communities (Figure 2). During the colonization process, overall community relatedness (NRI) was not affected by habitat size at the invertebrate scale but was highest at intermediate bromeliad sizes within the Insecta and Diptera scales. In turn, neighbor species in the colonizing communities were—significantly or marginally—more related in smaller bromeliads at all three taxonomic scales. During the extinction process, both relatedness metrics decreased with habitat size—that is, larger tanks contained, on average, species that are more distantly related than smaller tanks. This effect was not statistically supported at the Insecta scale, but at the Diptera scale NTI also decreased with habitat size (Figure 2; Appendix S1: Table S2).

**FIGURE 4:**
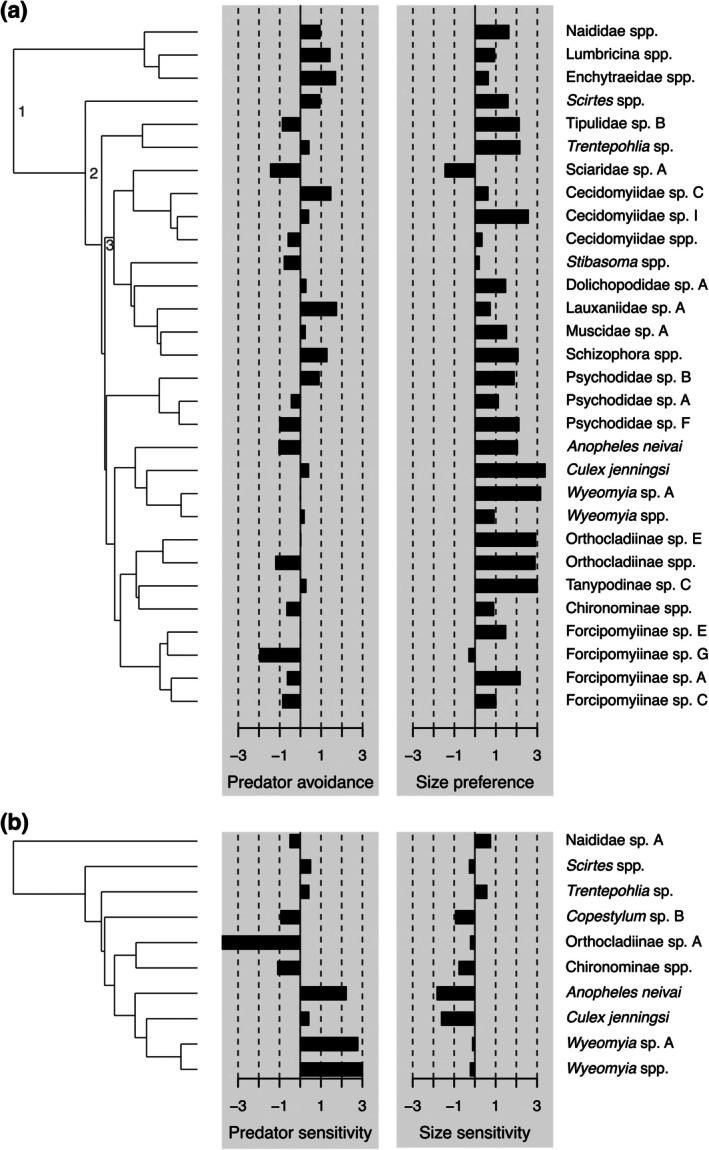
Distribution of traits across the community's phylogeny. Bars represent standardized response indices (*z*‐scores) quantifying deviations of observed species distributions across experimental treatments from the mean expected under a null assembly model. (a) Predator avoidance and size preference during colonization. Positive values indicate species that, relative to mean random expectations, colonized less frequently in bromeliads with predators or were distributed in larger bromeliads, respectively. (b) Predator and size sensitivity in the extinction experiment. Positive values indicate species that, relative to the null model mean, survived less frequently in bromeliads with predators or survived in larger habitats, respectively. Each experiment had a different species pool. For the colonization experiment, we only included species with more than one occurrence in the analyses. Nodes with numbers correspond to different taxonomic scales of analysis: 1 = Invertebrates, 2 = Insecta, 3 = Diptera.

Colonizing species generally showed a preference for large bromeliads (Appendix S1: Figure S5); that is, the mean bromeliad size in which individuals of a species were found was higher than expected if colonization across the size gradient was random (Appendix S1: Figure S5). As the 12 species with significant habitat size preference were distributed among seven different Diptera families, this trait did not show a statistically significant phylogenetic signal either across the entire invertebrate community or for internal clades (Appendix S1: Table S3). From the extinction experiment, we derived sensitivity indices for habitat size for each species in our designed communities, which compare the mean bromeliad size of persisting individuals with that expected under random persistence. Due to the limited number of prey morphospecies (10), we could not formally measure phylogenetic signals for these traits. No species had a significant size sensitivity index (all *z*‐scores <|1.96|), nor were there obvious phylogenetic patterns (Figure 4; Appendix S1: Figure S6).

### Predator effects

In natural communities, predator biomass did not affect prey species richness. However, predator biomass was negatively associated with the overall relatedness of the invertebrate community (NRI; Figures 2 and 3)—that is, prey species were more distantly related in bromeliads with higher predator biomass. This pattern persisted at the dipteran scale, but not at the insect scale. Our experiments showed that predators affected species richness via extinction and relatedness via colonization (Figure 2; Appendix S1: Table S2). In the extinction experiment, predators substantially reduced species richness at all taxonomic scales, but did not affect relatedness metrics. In the colonization experiment, by contrast, predators did not affect species richness metrics but increased the relatedness of close relatives (NTI) at the invertebrate scale (although not at narrower scales).

The effects of predators on prey relatedness during colonization may reflect an underlying phylogenetic signal in predator avoidance. We compared, using *z*‐scores, the proportion of a species’ colonists found in predator‐free bromeliads to the proportion predicted if bromeliad choice was arbitrary with respect to predator presence. Although the predator avoidance indices for most species individually fell within the 95% confidence bounds for the null model (Appendix S1: Figure S5), collectively, predator avoidance showed a significant yet moderate phylogenetic signal across the invertebrate community (Appendix S1: Table S3). This invertebrate‐wide signal may reflect avoidance of predators (positive indices) for all three Annelida taxa, evolutionarily distant from the remaining taxa, which are all Insecta, coupled with positive associations (negative indices) with predators within Diptera families like Ceratopogonidae (Figure 4). Narrower taxonomic scales, however, did not exhibit a significant signal (Appendix S1: Table S3).

Although we did not have enough species in the extinction experiment to similarly test for a phylogenetic signal, we computed a predator sensitivity index, using *z*‐scores, that compares the proportion of persisting individuals in predator‐free bromeliads to that expected if individuals persisted at random. Many prey species did not have predator sensitivity indices that differed from community‐wide null expectations (Appendix S1: Figure S6), although *Orthocladiinae* chironomids were significantly less sensitive than expected, and three of the four Culicidae species (two *Wyeomyia*, one *Anopheles*) were significantly more sensitive than expected. The fourth Culicidae species, *Culex* sp., was much less sensitive than *Anopheles* and *Wyeomyia* species (Figure 4; Appendix S1: Figure S6).

## DISCUSSION

By combining natural surveys of invertebrate communities with experiments that isolated colonization or extinction processes, we tested hypotheses on how two key environmental drivers, habitat size and predator presence, affect species richness and relatedness of invertebrate prey. We found that an observed increase in invertebrate species richness with habitat size in natural communities was largely driven by colonization processes: More species preferred larger bromeliads during colonization. In contrast, effects of predators on species richness were driven largely by extinction processes: Predation resulted in the loss of established invertebrate species from the community. These patterns in species richness, whether from surveys or experiments, were robust across a range of taxonomic scales. Conversely, patterns of relatedness with habitat size and predator biomass were dependent on the taxonomic scale, and natural patterns often did not match tendencies found in experiments isolating colonization and extinction processes.

### Habitat size

The consistent positive effect of habitat size on species richness in natural and experimental colonizing communities may be related to the influence of habitat size on the amount of biological life that can be supported within a single bromeliad. Previous work has shown that larger bromeliads have more stable environments, more detrital resources, and support more individuals, all of which may be linked to their higher species richness (Amundrud & Srivastava, 2015; Guzman et al., 2019; Srivastava, Ware, et al., 2020). By separating colonization and extinction processes, we observed that the effects of bromeliad size on prey species richness and relatedness were largely determined through colonization preferences, possibly reflecting habitat selection by dispersing adults or effects of habitat size on habitat detectability. We expected extinction to amplify these colonization effects because small bromeliads are more prone to drought and associated species loss and filtering. However, experimental bromeliads were continuously water‐filled as our study was conducted during the rainy season to ensure high diversity; extinction processes may be more important in the dry season when small bromeliads are at greater risk of drying out (Amundrud & Srivastava, 2015; Srivastava, Ware, et al., 2020). Nonetheless, colonization preferences by adult insects are likely influenced by the expectation of potential drought in small bromeliads, especially for species with long larval stages that extend into the dry season (Guzman et al., 2019). Indeed, when species exhibited strong size preferences, these were all for larger bromeliads. Habitat selection according to patch size has been observed in other aquatic invertebrates; for instance, species on intertidal rock platforms generally select larger patches (Matias et al., 2010), whereas in pond systems, the preferred patch size varies among and within major taxa (Resetarits et al., 2019).

Compared to the consistent effects of habitat size on species richness, the effects on relatedness metrics were more variable. In natural communities, the effect of habitat size on overall relatedness (NRI) switched from positive at the invertebrate scale, to a quadratic relationship within the insect community, to negative or nonexistent within dipterans or among close relatives (NTI), suggesting that bromeliad size may act as a mediator for competition at broad taxonomic scales while acting mainly as a habitat filter at narrow taxonomic scales. Such effects of taxonomic scale can be explained by the full community containing lineages with distinct evolutionary histories—that is, phylogenetically divergent taxa with potentially distinct life histories. In communities with unbalanced trees—such as the one in this study (Appendix S1: Figure S2)—where a few deep lineages have long branches and few close relatives, these deeply diverging taxa can disproportionately affect phylogenetic metrics and mask the patterns in the rest of the community, even when they are not a dominant clade (Mazel et al., 2016). In this case, the broadest taxonomic scale of invertebrates is the only one to include annelids, an evolutionarily divergent group that, unlike the rest of the prey community, reproduces within bromeliads. This leads to the potential for multigenerational population dynamics among competing species. Previous theory has predicted relatedness to increase with habitat size because larger habitats encompass higher environmental heterogeneity, allowing the coexistence of many related species (Cavender‐Bares et al., 2009; Helmus & Ives, 2012). This hypothesis has received mixed support from vascular plants (Matthews et al., 2020), but to the best of our knowledge has not been evaluated for invertebrate communities. Although this hypothesis was formulated for larger spatial scales where species interactions are weak and macroecological and macroevolutionary processes (e.g., dispersion, in situ speciation) are involved, it may also apply to multigenerational species like annelids in bromeliads where subdivision of the aqueous habitat by leaves weakens species interactions, especially in large plants (Srivastava, 2006). By contrast, for insect species that spend only their larval stage in bromeliads, competition may be less important than the filtering effects of small habitat size on species sensitive to drought. For example, most species in the Culicidae family, a dominant dipteran family in the system, are particularly sensitive to fluctuating water levels (Dézerald et al., 2015; Srivastava, Céréghino, et al., 2020).

Although our surveys suggest that bromeliad size mediates competition at broad taxonomic scales and acts mainly as a habitat filter at narrow scales, there were important mismatches in relatedness patterns between the surveys and experiments, especially at the invertebrate scale. Mismatches between observations and experiments are often caused by differences between short‐term and long‐term community dynamics (Connell & Sousa, 1983), and we suggest this is occurring here. Annelids—included only at the invertebrate scale—rely on phoretic and passive dispersal (Lopez et al., 2005), which may happen infrequently. Because of the limited time of our colonization experiment, only a few bromeliads were colonized by annelids and rarely by more than one species. By contrast, annelids are present in virtually all natural bromeliads, with multiple species often present. Similarly, in the extinction experiment, we included only one annelid species, preventing us from perceiving the effects of interactions among annelids. These discrepancies are consistent with findings from studies of ecological succession in other systems, where phylogenetic relatedness often changes across successional stages, although the direction varies across studies and taxa (e.g., Brown & Jumpponen, 2015; Chai et al., 2016; Purschke et al., 2013). However, the effects of habitat size on close relatives (NTI) were more consistent than on overall relatedness (NRI), with NTI tending to be negatively related to habitat size. Relatedness among close relatives (NTI) is less sensitive to the influence of long branches (Molina‐Venegas & Roquet, 2014) than overall relatedness (NRI) and better captures variation near the tips of the phylogeny. Therefore, the inclusion of phylogenetically distinct groups in the analyses (i.e., broader taxonomic scales) or differences in experimental settings (i.e., surveys including more individuals and occurrences of annelids) did not influence NTI patterns as strongly as NRI, indicating that at narrow taxonomic scales, small bromeliads often lead to clustered communities through both colonization and extinction effects.

### Predator effects

Our experiments show that predators cause taxonomically widespread reductions in species richness through reducing survival rather than colonization. Such reductions in diversity are consistent with the known strong consumptive effects of damselfly predators in this system (De Omena et al., 2019; LeCraw & Srivastava, 2019). By contrast, we did not detect non‐consumptive: In the colonization experiment, the mere presence of predators did not affect the number of individuals, species richness, or phylogenetic relatedness that colonized each plant. This lack of non‐consumptive on colonization is surprising, given a previous study demonstrating non‐consumptive effects of the same predatory damselfly on colonization of specific bromeliad invertebrates (Hammill, Atwood, and Srivastava 2015), and given widespread documentation of predator‐induced habitat selection for taxa with aquatic development, from amphibians to insects (Binckley & Resetarits, 2005; Blaustein, 1999; Resetarits et al., 2019; Resetarits & Wilbur, 1989). Our results instead suggest that, for prey in our system and under our experimental conditions, perceived drought risk or habitat complexity may be stronger determinants of habitat quality than risk of predation. Furthermore, the strong top‐down consumptive effects seen in the extinction experiment were not apparent in natural communities, perhaps because these consumptive effects are obscured by rapid colonization, which replace in part the individuals and species eliminated by the predator, as well as by the eventual emergence of adult insects.

Similar to habitat size effects, the effects of predators on prey relatedness were not concordant between natural and experimental bromeliads. In natural communities, higher biomass of predators was associated with more dispersed (less related) prey communities at either the Invertebrate or Diptera scale. A similar dispersed pattern has been reported for macroinvertebrate communities under fish predation in rivers, in this case due to preferential consumption by fish of insects (Helmus, Mercado‐Silva, and Vander Zanden 2013). In our study, such dispersed patterns are contrary to our initial expectations based on conservation of predator avoidance or vulnerability traits. Dispersed prey communities are expected if prey response to predators is evolutionarily convergent; that is, if a variety of different traits enable predator detection and avoidance. Accordingly, we found a moderate to low signal for predator avoidance during colonization, detected only at the invertebrate level.

During colonization, predators increased relatedness among invertebrate close relatives. This could be driven by a sixfold reduction in annelid colonization in bromeliads with predators, leading to increased relatedness among close relatives (NTI). As discussed earlier, annelids are phylogenetically distant from the rest of the community and the only taxa reproducing within bromeliads, so their inclusion can strongly influence relatedness patterns. Annelid colonization—at least for some species—happens through phoretic dispersal (Lopez et al., 2005); therefore, the higher frequency of colonizers in bromeliads without predators might be mediated by the phoretic host avoiding predatory damselfly larvae. The mismatch with relatedness patterns in natural bromeliads may reflect the short time frame of the colonization experiment, restricting the potential number of phoretic introductions of annelids and growth of annelid populations, and thus limiting competition pressure between annelid species. In addition, the negative effects of predators on relatedness in natural bromeliads may also reflect differences between related species in their vulnerability to predation, as exemplified by divergent sensitivities of Culicidae species in the extinction experiment (Figure 4; Appendix S1: Figure S6). Here, we found *Culex* mosquitoes survived co‐occupancy with predatory damselflies better than *Anopheles* and *Wyeomyia* mosquitoes. This result is consistent with documented differences between mosquito taxa in their ability to avoid detection by damselflies through behavioral modification (Hammill, Atwood, Corvalan, & Srivastava, 2015). Although many morphological traits are conserved at the family or higher level in aquatic invertebrates, behavioral traits are more labile and can effectively reduce predation (Welborn et al., 1996; Werner & Peacor, 2003).

## IMPLICATIONS

Our study is novel in applying a patch dynamic approach to understand the drivers of phylogenetic diversity in ecological communities. By experimentally separating colonization and extinction processes and comparing their effects with the net effects observed in natural communities, we were able to reveal the ecological and evolutionary mechanisms underlying phylogenetic community structure. We advocate for such an integrated approach in phylogenetic community ecology in general, as conceptual progress requires empirical tests of theoretically supported mechanisms. Although previous studies have considered phylogenetic signals in species colonization rates, species extinction rates, and trophic interactions separately, a truly mechanistic theory of phylogenetic community ecology should integrate these processes at temporal and spatial scales relevant to multitrophic communities. A third important scale here is taxonomic: Our study illustrates the value of examining patterns at multiple taxonomic scales, as traits involved in different ecological processes may potentially originate from different depths in the phylogenetic tree. Understanding the dependence of patterns on the taxonomic scale may therefore help reconcile previously divergent results between studies.

## AUTHOR CONTRIBUTIONS

Nadia B. Páez‐Rosales, Diane S. Srivastava, and Jessica L. Ware designed the research. Nadia B. Páez‐Rosales performed the experiments, data collection, and analyses. Nadia B. Páez‐Rosales and Diane S. Srivastava wrote the paper. Jessica L. Ware revised the paper.

## CONFLICT OF INTEREST STATEMENT

The authors declare no conflicts of interest.

## Supporting information


Appendix S1.

## Data Availability

Data and code (Páez‐Rosales et al., 2026) are available in Dryad at https://doi.org/10.5061/dryad.31zcrjdw7.
