# Supplementary material for: Colonization and extinction mediate environmental effects on the phylogenetic diversity of invertebrate communities
Source: Ecology. 2026 May 8;107:e70400. doi: 10.1002/ecy.70400 (PMC13156477; doi:10.1002/ecy.70400)
Supplement: Supplementary file 1 — Appendix S1. [file ECY-107-e70400-s001.pdf]

Appendix S1

Colonization and extinction mediate environmental effects on the phylogenetic diversity of invertebrate communities

Nadia B. Páez-Rosales, Jessica L. Ware, Diane S. Srivastava

Ecology

Section S1. Supplementary Methods

Species delimitation

To detect cryptic diversity, we extracted the DNA and sequenced the barcoding region of the Cytochrome c oxidase subunit I (COI) from three individuals per morphospecies, when available. We reconstructed a preliminary maximum-likelihood phylogeny from the community's COI sequences and used a Poisson Tree Process (PTP) analysis on the additive tree to delimit putative species. For any morphospecies that PTP split into more than one Operational Taxonomic Unit (OTU), we sequenced additional specimens and re-ran the analyses with the extended dataset to refine the delimitation. As a result, a total of 246 specimens were sequenced for COI.

The alignment of the COI matrix was performed in Mesquite v. 3.61 (1) with the MAFFT (L-INS-i) algorithm, and the ML tree was reconstructed with RAxML v 8.2.12 (2). We set 5 replicates with three partitions, one for each codon position, and used the GTRGAMMAI model. For support values, we run 1,000 bootstrap samples with similar settings. The PTP analysis for our rooted COI tree was conducted on the web server https://species.h-its.org/ptp/ (3) with the default settings. The PTP approach recognized the presence of 128 Operational Taxonomic Units in the community. Since phylogenetic and molecular information are not standalone evidence for species delimitation, we considered morphology to support the delimitation proposed by the PTP analyses. We recognized 124 species from the delimitation analyses, which represents double the diversity previously reported for the field site's species pool. We additionally sequenced the 28S-D8 marker for subsequent analyses.

For DNA extractions, we used Qiagen's DNeasy Blood & Tissue Kit. For amplification of the COI, we used the universal primers LCO-1490 (5'-GGTCAACAAATCATAAAGATATTGG-3') and HCO-2198 (5'-TAAACTTCAGGGTGACCAAAAAATCA-3') or RON (5'-GGATCACCTGATATAGCATTCCC-3') and NANCY (5'-CCCGGTAAAATTAAAATATAAACTTC-3') when the former pair failed. To amplify the D8 region of the 28S, we used the primers FLalt1 (5'-GTAGGTAAGGGAAGTCGGC-3') and GALT (5'-TGTCTCCTTACAGTGCCAGA-3'). The PCR cycle settings for both markers were: an initial denaturation step at 94˚C for 3 min, followed by 35 amplification cycles (denaturation at 94˚C for 30 sec, annealing at 50˚C for 30 sec, and elongation at 72˚C for 45 sec), and a final elongation step at 72˚C for 10 min. PCR products were sent to Psomagen (Rockville, MD, USA) for sequencing.

Phylogenetic reconstruction

We built a time-calibrated species phylogeny of the community to extract phylogenetic diversity metrics. For this, we sequenced a mitochondrial and a nuclear marker for each species, the barcoding region of the COI and the D8 region of the 28S, respectively, chosen for their availability in GenBank. The use of two markers was due to budget constraints. This community encompasses species from divergent

taxa, even from different Superphylla (e.g., Spiralia vs. Ecdisozoa), and the information from two markers is not enough to recover these ancient divergences. Several studies have tried to infer deep relationships in the invertebrate phylogeny from morphologic, genomic, or proteomic information (e.g., 7–9), including a broader and more even taxonomic sampling than our study. Even then, many branching events are not well-resolved. To address this problem, we incorporated available information by using topological constraints in our analyses based on the Open Tree of Life synthesis phylogeny (7) and on recent systematic studies not yet included in the Open Tree of Life.

Our dataset included 124 taxa from our community, plus 80 taxa added from GenBank to fill taxonomic gaps and provide taxonomic references for the main taxa. Each gene was aligned in Mesquite v. 3.61 with the MAFFT (L-INS-i) algorithm, followed by a manual alignment. We used only the first and second codon positions of the COI (438 bp) and excluded non-homologous regions in the 28S (445 bp), leaving us with an 883-bp alignment. We inferred a dated Bayesian phylogeny using BEAST2.7.5 (8) and associated packages, incorporating fossil information to calibrate 39 nodes. We specified three partitions (first and second codon positions of the COI and the 28S), each with an optimized relaxed clock, and let the site model be simultaneously estimated with BModelTest (9). The tree was inferred under a birth-death model, and we added priors for node ages and topological constraints (Figure S3, Table S1). For time calibrations, we used a uniform distribution, setting the minimum (earliest fossil available for a clade) and maximum boundaries. For details on fossil calibrations, see Table S1. We ran four independent MCMC chains for 50M generations, and sampled trees every 5000 generations. We analyzed convergence and Effective Sample Size with Tracer v1.7 (10), and performed parallel runs sampling only from priors to ensure our data was informative. With LogCombiner, we discarded 100 trees from each chain as a burn-in, combined the remaining trees and thinned our samples by resampling every 20000 generations, resulting in a total of 9004 trees. We used TreeAnnotator to get the maximum clade credibility tree and its associated statistics.

Species traits and phylogenetic signal

We used species' responses to experimental conditions of habitat size and predator presence as proxies for underlying traits. To measure these responses, we developed indices based on species distribution across treatments separately for colonization and extinction experiments. From the colonization experiment, we derived indices of predator avoidance and habitat size preference based on the distribution of colonizing species, while from the extinction experiment, we derived indices of predator sensitivity and size sensitivity based on the distribution of surviving individuals across treatments. For all responses, we captured the deviation of the observed distribution of each species from null expectations using *z-scores*. For predator response indices, we calculated the *z-score* for the proportion of individuals in predator-free communities relative to null expectations, such that positive values indicate species occurring more frequently in predator-free bromeliads than expected under the null mean. For bromeliad size response indices, we calculated z-scores for the mean bromeliad size (log-scale) in which individuals of a species were found, with positive values indicating an association with larger bromeliads relative to the null mean.

To build null communities for the colonization experiment, we shuffled bromeliads (rows) across species (columns) in a matrix summarizing the composition of colonizing communities 1000 times. Such a process simulates random colonization of bromeliads by local populations, as expected when insects oviposit large egg clusters. We reran this null model with random colonization by individuals (e.g., single egg oviposition) and obtained qualitatively similar results. To build null communities for the extinction experiment, we randomly sampled (1000 times with replacement) the identities of individuals in each bromeliad until reaching the total abundance at the end of the experiment. The probability of sampling a given species was equal to its initial frequency, and the null model simulates stochastic mortality at the individual level.

We assessed phylogenetic signal only for colonizing communities since the species pool of the extinction experiment was too small for reliable calculations. For colonizing communities, we included only species occurring in more than one community. To measure and locate the phylogenetic signal of the size preference and predator avoidance indices, we used the *phyloSignal* and *phyloSignalINT* functions from the *phylosignal* package, estimating *Moran's I*, *Abouheif's Cmean*, *Pagel's λ*, and *Blomberg's K* for nodes corresponding to the entire community and Insecta and Diptera subsets. Cryptic species, unknown before the experiments, were collapsed into a single tip and assigned presence and abundance values for the morphospecies.

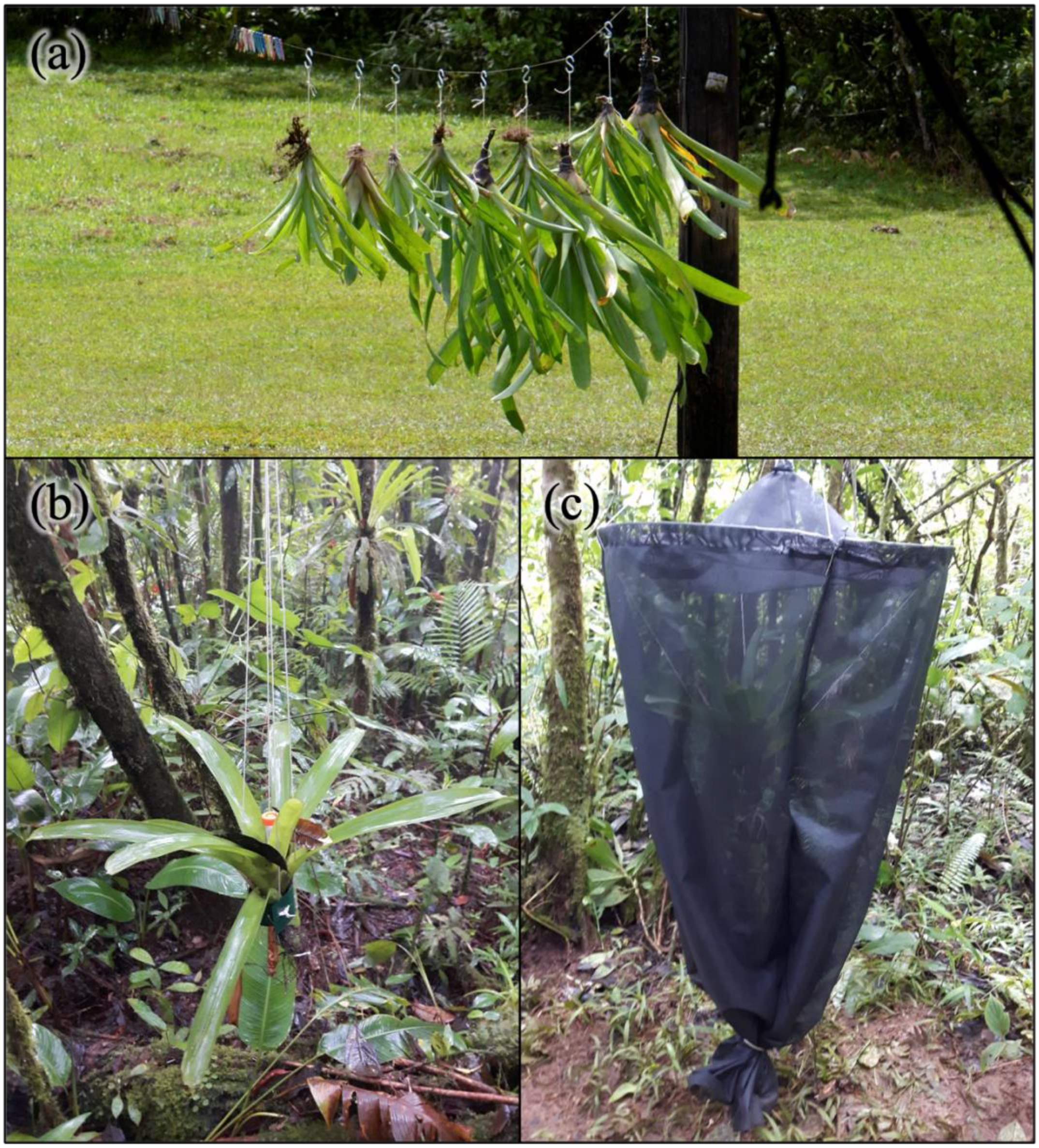

Figure S1. Field experimental setup. **(a)** Drying process after emptying and cleaning the bromeliads before the experiments. **(b)** Set up of the colonization experiment. Each bromeliad was refilled with standardized amounts of water and detritus scaled to its size. Half of the bromeliads received a predator treatment consisting of damselfly larvae (number scaled to bromeliad size), each placed in a tube with small holes covered with a mesh; predator-absent treatments contained empty tubes. **(c)** Set up of the extinction experiment. Each bromeliad was refilled with water, detritus, and an initial prey community composed of ten species, with abundances scaled to bromeliad size. Free damselfly larvae were placed in half of the bromeliads. Photo credits: (a) Diane S. Srivastava and (b, c) Nadia B. Páez-Rosales.

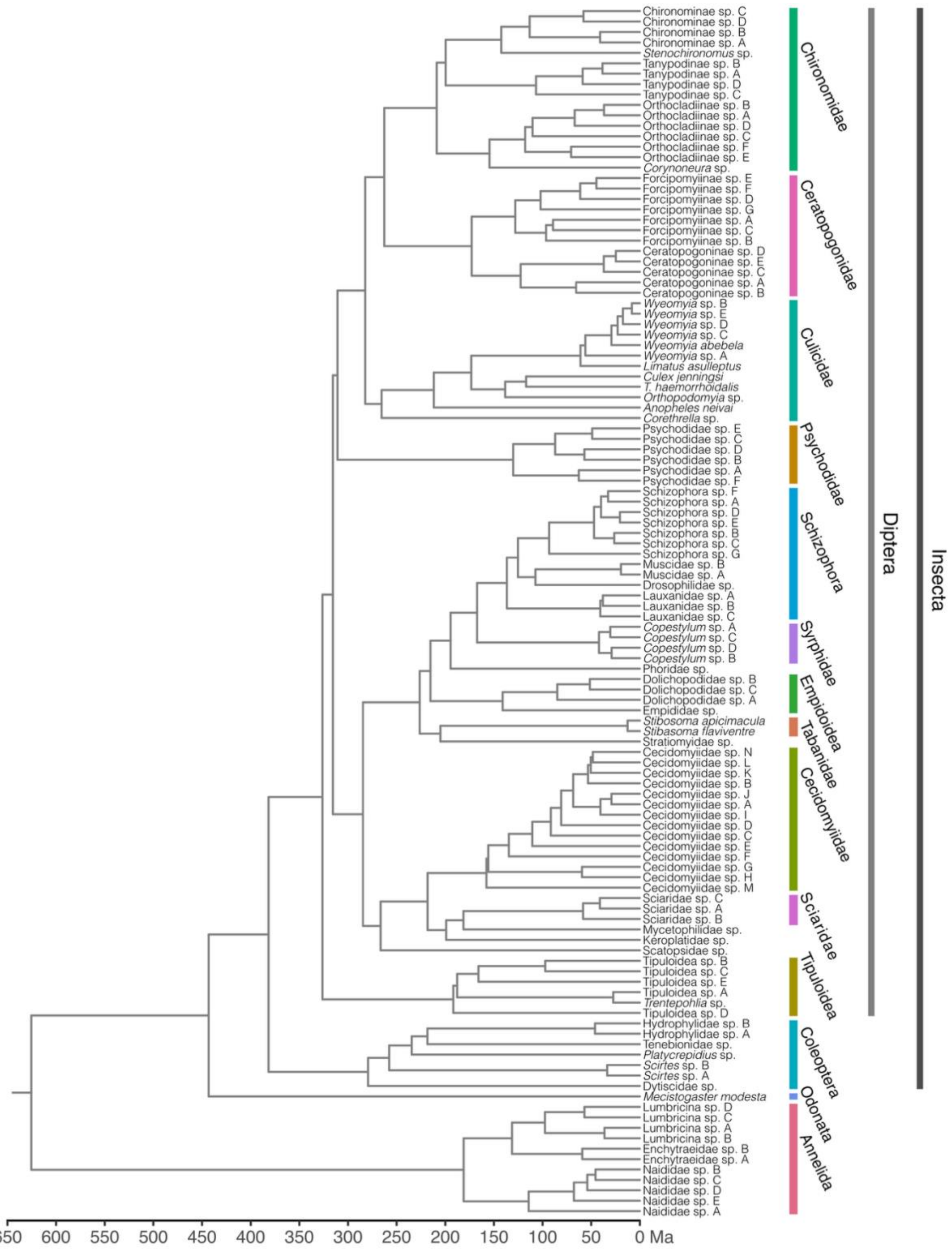

Figure S2. Time-calibrated phylogeny for the community of aquatic invertebrates in bromeliads. The phylogeny includes aquatic and semiaquatic species in the regional pool of Pitilla Biological Station, Costa Rica; other taxa are trimmed. See Figure S3 for a phylogeny with all the tips included in the analyses and confidence intervals for time estimations. Details on node calibrations are given in Table S1.

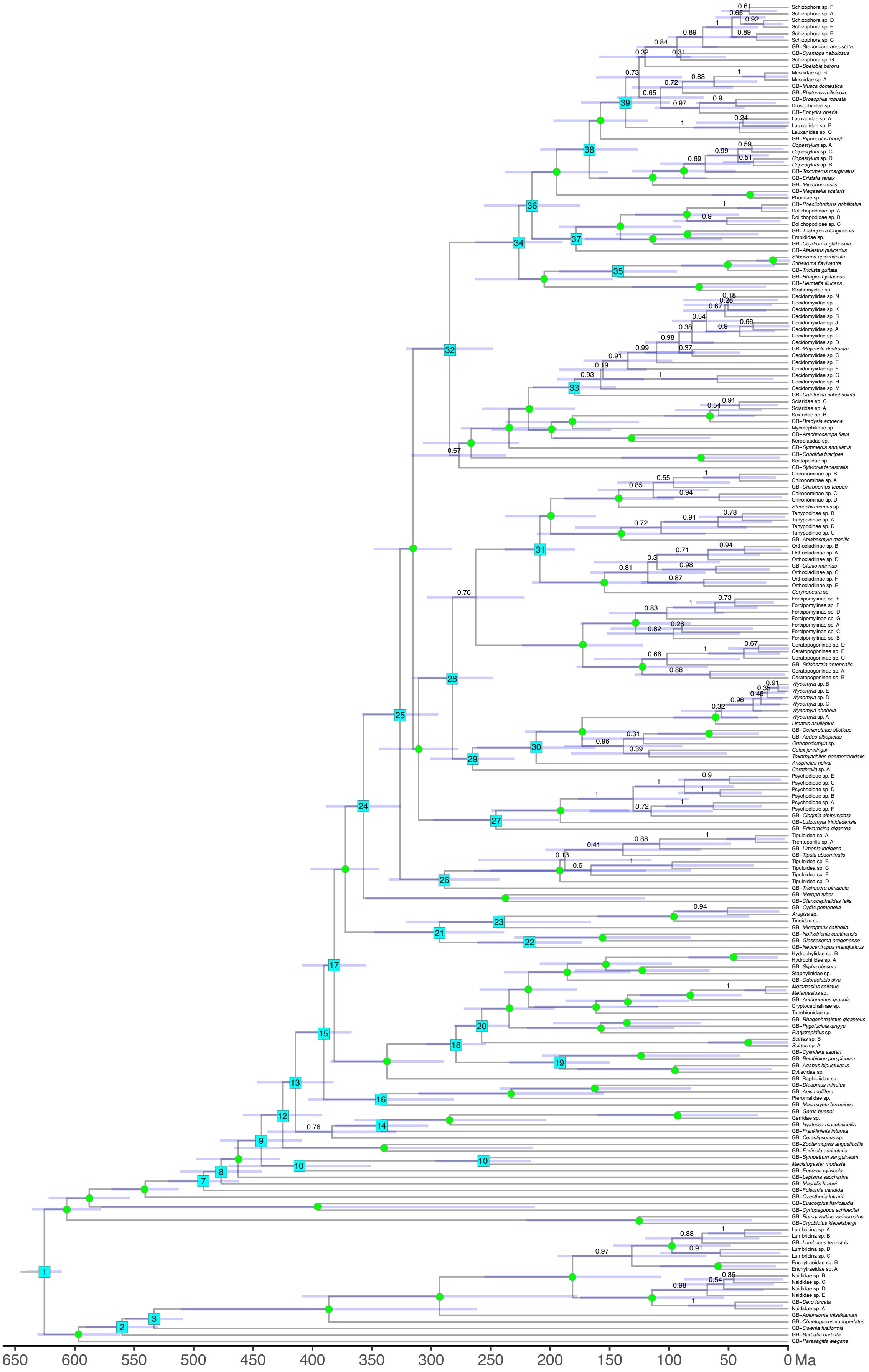

Figure S3. Time-calibrated phylogeny of the aquatic invertebrate community associated with bromeliads, including taxa external to the bromeliad system used for phylogenetic inference. Maximum clade credibility tree from BEAST2 analyses inferred from COI and 28S genes. Sequences from GenBank are marked with GB. Blue bars represent 95% confidence intervals for age estimates, and numbers above branches represent posterior probabilities. Topological constraints (green circles) and time calibrations (blue squares) were applied to selected nodes; node numbers correspond to calibration information in Table S1.

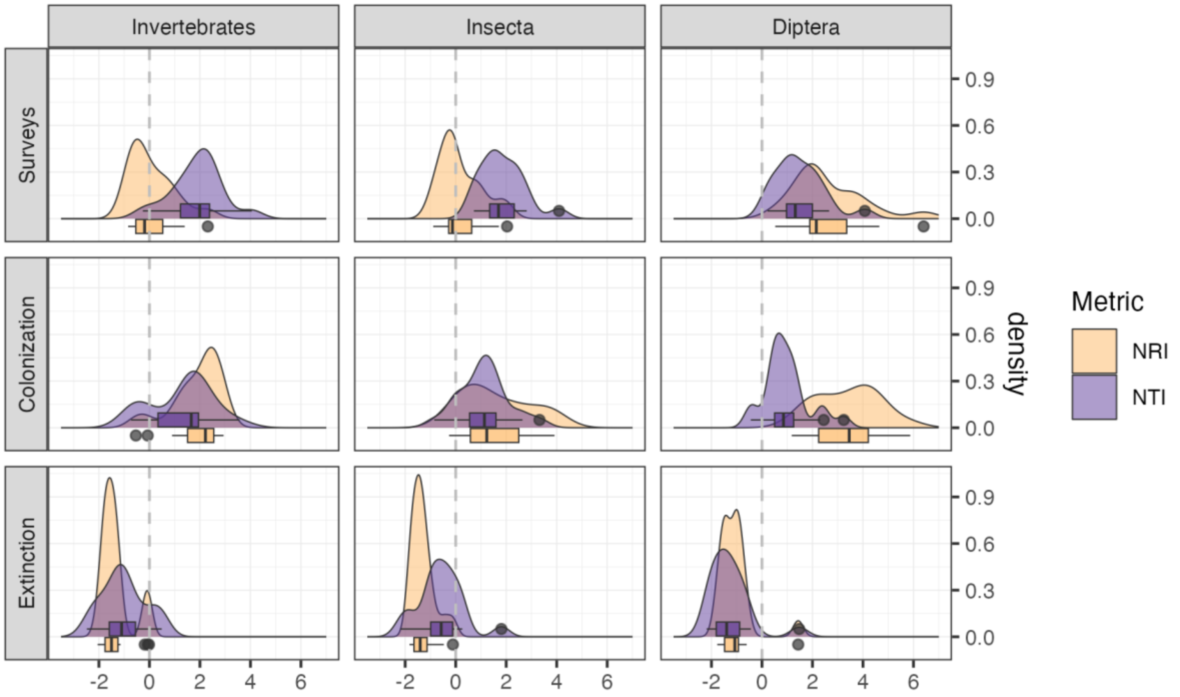

Figure S4. Distributions of phylogenetic community relatedness metrics across experimental contexts and taxonomic scales. Density curves and embedded boxplots show values of the net relatedness index (NRI) and nearest taxon index (NTI) for communities evaluated at three taxonomic scales, and across survey, colonization, and extinction datasets.

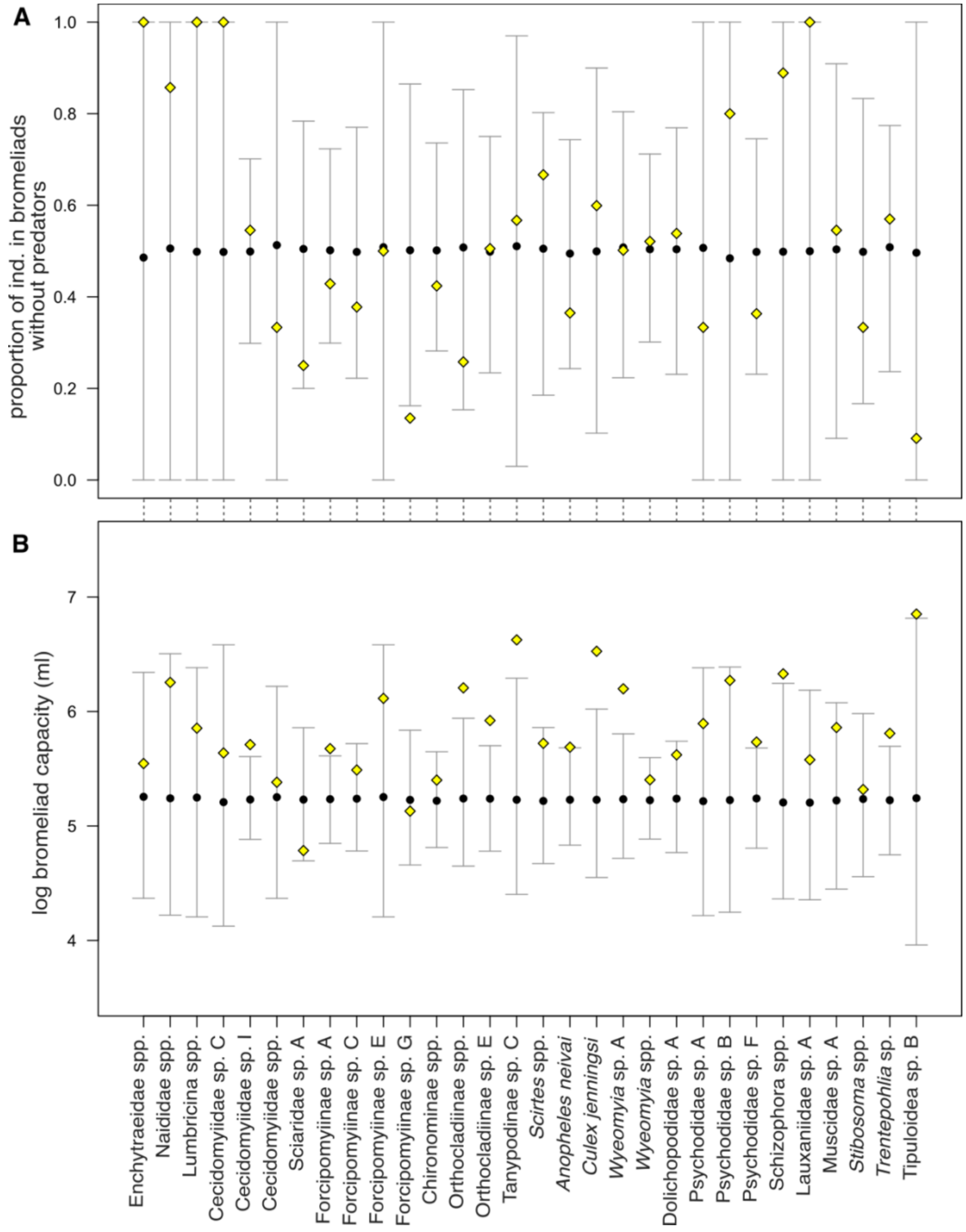

Figure S5. Observed versus expected distribution of species across environmental conditions in the colonization experiment. **A.** Observed proportion of individuals of each species found in bromeliads without predators (yellow diamonds) against 95% confidence interval for proportion predicted with null model for random colonization, and their mean (black dots). **B.** Observed mean bromeliad size for individuals of each species (yellow diamonds) against 95% confidence interval for mean size predicted with null model for random colonization and their mean (black dots).

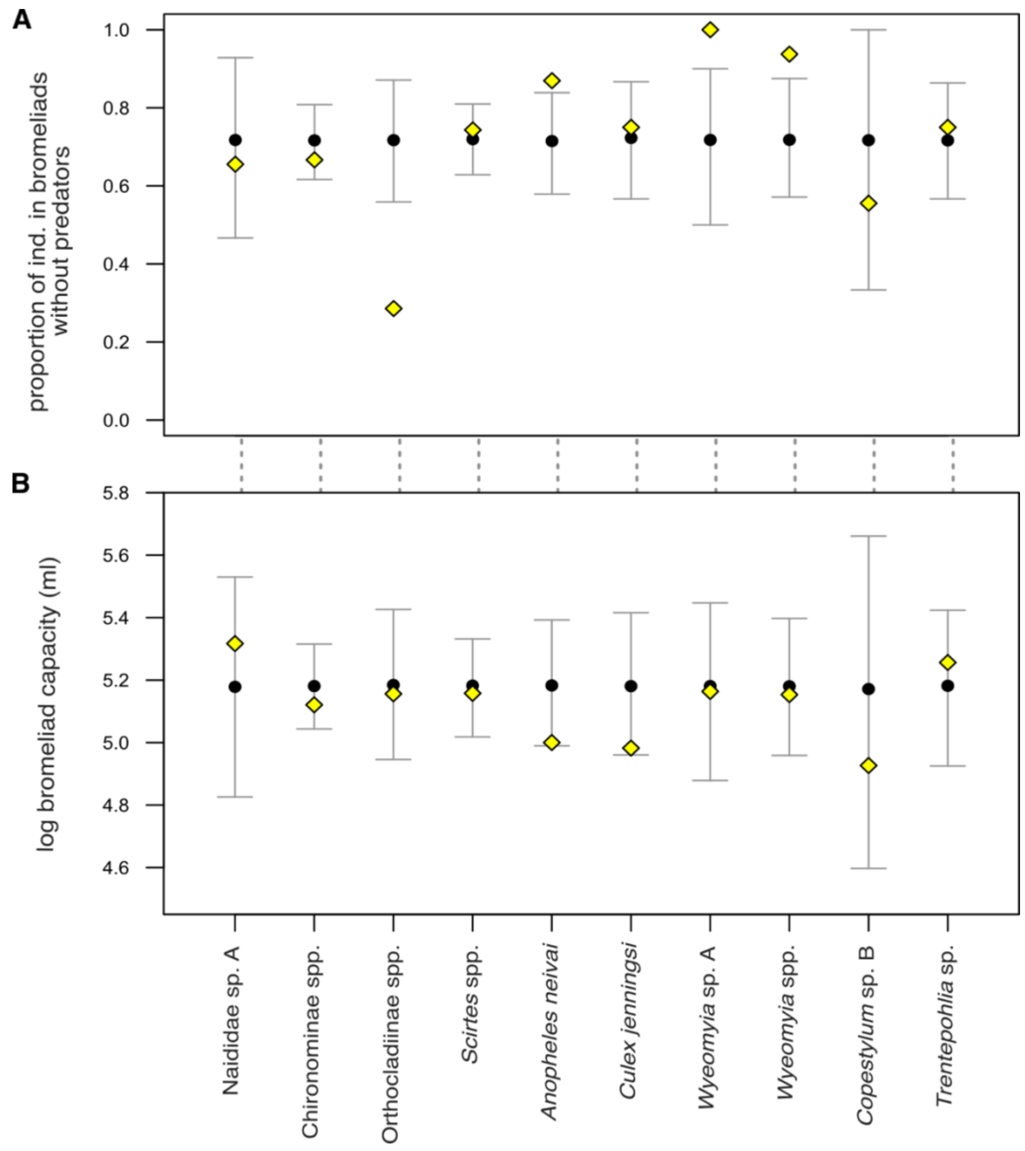

Figure S6. Observed versus expected distribution of species across environmental conditions in the extinction experiment. **A.** Observed proportion of individuals of each species found in bromeliads without predators (yellow diamonds) against 95% confidence interval for proportion predicted with null model for random extinction and their mean (black dots). **B.** Observed mean bromeliad size for individuals of each species (yellow diamonds) against 95% confidence interval for mean size predicted with null model for random extinction and their mean (black dots).

Table S1. List of fossils used for node calibrations. This table presents the fossil species used for each node, specimen ID when available, their assigned clade in our phylogeny, and minimum and maximum age constraints. We used a uniform distribution for all calibrations. Node number corresponds to those in Figure S3. Sources for fossil placement and age settings are provided.

Node	Specimen ID	Fossil	Group assignment	Minimum age [Ma]	Maximum age [Ma]
1		*Kimberella quadrata* (11)	Protostomia (crown)	550.25	636.1
2		*Aldanella yanjiahensis* (11)	Annelida+Mollusca (crown)	532	636.1
3	YKLP 11382	*Dannychaeta tucolus* (12)	Annelida (crown)	514	636.1
4		*Rusophycus avalonensis* (11, 13)	Ecdysozoa (crown)	528.82	636.1
5	YKLP 10840	*Yicaris dianensis* (11, 13)	Euarthropoda (crown)	514	636.1
6	YKLP 10840	*Yicaris dianensis* (13)	Altocrustacea (crown)	514	636.1
7	NHMUK IN. 27765	*Rhyniella praecursor* (13)	Hexapoda (crown)	405	521
8	BMNH IN. 38234	*Rhyniognatha hirsti* (13)	Insecta (crown)	405	521
9	BGR x 9216	*Delitzschala bitterfeldensis* (13)	Pterygota (crown)	322.83	521
10	CNU-NX2006003	*Oligotypus huangheensis* (13)	Palaeoptera (crown)	319.9	521
11	MACN No. 18040,	*Triassothemis mendozensis* (13)	Odonata (crown)	228	521
12	IRSNB a9885	*Protoprosbole straeleni* (13)	Neoptera (crown)	319.9	521
13	IRSNB a9885	*Protoprosbole straeleni* (13)	Eumetabola (crown)	319.9	521
14	Avion No. 2	*Aviorrhyncha magnifica* (13)	Condylognatha (crown)	306.9	411
15	MNHN-LP-R.55181	*Westphalomerope maryvonneae* (13)	Holometabola (crown)	313.7	411
16	PIN 2070/1	*Triassoxyela foveolata* (13)	Hymenoptera (crown)	226.4	411
17	MNHN-LP-R.55181	*Westphalomerope maryvonneae* (13)	Aparaglossata (crown)	313.7	411
18	AM F40278	*Ponomarenkium belmonthensis* (14)	Coleoptera (crown)	251.878	307.1
19	*PIN 5381/32*	*Tunguskagyrus planus* (14)	Dytiscidae + Carabidae (crown)	155	251.878
20	NIGP162053	*Elateriformia* sp. (14)	Polyphaga (crown)	237	293.69
21	BMNH In. 59397	*Archaeolepis mane* (13)	Amphiesmenoptera (crown)	195.31	411
23	LGA 1995, LGA 672, LGA 1710	*Liadotaulius maior* (13)	Trichoptera (crown)	179.66	411
22		*Parasabatinca aftimacrai* (13)	Lepidoptera (crown)	129.41	411
24	PIN966/21	*Pseudonannochorista willmanni* (13)	Antliophora (crown)	268.3	411
25	No. 5514	*Grauvogelia arzvilleriana* (13)	Diptera (crown)	240.5	411
26	SMNS, No. 9052	*Archilimonia grauvogeliana* (15, 16)	Tipulomorpha (stem)	240.5	411
27	LGA 1145	*Nannotanyderus krzeminskii* (17–19)	Psychodomorpha (crown)	183.7	411
28	Larva 6187a	*Anisinodus crinitus* (16, 20, 21)	Culicomorpha (crown)	240.5	411
29	PIN 5329/12	*Triassomyia shcherbakovi* (22, 23)	Culicidae + Chaoboridae (crown)	227.2	411
30	Poinar B-D-70	*Priscoculex burmanicus* (21, 24, 25)	Anophelinae (stem)	98.17	411
31	LGA 835	*Oryctochlus toarciensis* (19, 26)	Chironomidae (crown)	183.7	411
32	No. 6171 (+,-)	*Gallia alsatica* (16, 27, 28)	Brachycera (stem)	240.5	411
33	PIN 3063/580	*Mesotrichoca mesozoica* (19, 29, 30)	Cecidomyiidae (crown)	142.5	411
34	PIN 358/120	*Oligophryne fungivoroides* (17–19)	Brachycera (crown)	192.6	411
35	AMNH Bu-SE2/10,	*Tabanipriscus transitivus* (25, 31)	Tabanomorpha (crown)	98.17	411
36	PI 3791/2859	*Protoreogeton admirabilis* (17–19)	Eremoneura (crown)	164.2	411
37	T54	*Dianafranksia fisheri* (17, 32)	Empidoidea (crown)	142.5	411
38	AMNH Bu-SE03	*Prosyrphus thompsoni* (25, 33)	Syrphidae (stem)	98.17	411
39	USNM 539754	*Phytomyzites biliapchaensis* (17, 34)	Schizophora (crown)	64	411

Table S2. Best-fit models for diversity metrics in natural and experimental communities across different taxonomic scales. Full models included habitat size (linear and quadratic terms), the predator variable, and their interaction. The predator variable was measured as predator biomass in the surveys and as predator presence in experiments. Habitat size corresponds to the logarithm of tank water capacity. Coefficients for each variable are followed by ANOVA II *p*-values in parentheses. "–" indicates that the variable was not selected in the best model, and (λ) indicates the response variable was transformed (Box-Cox or Yeo-Johnson) to meet linear model assumptions.

Dataset	Scale	Model	R^2 adj.	Habitat size	Habitat size^2	Predator biom/pres
Surveys	Invertebrate	Species Richness	0.28	0.5 (1.66e-02)	–	–
		NRI (λ)	0.37	0.82 (6.21e-03)	–	-0.06 (9.23e-03)
		Abundance (λ)	0.37	0.56 (5.58e-03)	–	–
	Insecta	Species Richness	0.35	0.55 (7.56e-03)	–	–
		NRI (λ)	0.36	4.85 (8.88e-03)	-0.47 (6.86e-03)	–
		wNRI (λ)	0.23	–	–	-0.03 (2.82e-02)
		Abundance (λ)	0.41	0.58 (3.47e-03)	–	–
	Diptera	Species Richness	0.35	0.55 (7.56e-03)	–	–
		NRI (λ)	0.35	–	–	-0.04 (7.64e-03)
		wNRI	0.25	–	–	-0.03 (2.50e-02)
		NTI (λ)	0.16	-0.4 (6.44e-02)	–	–
		Abundance (λ)	0.35	0.55 (7.08e-03)	–	–
Colonization	Invertebrate	Species Richness (λ)	0.53	0.83 (1.60e-04)	–	–
		NTI	0.32	-0.47 (3.83e-02)	–	0.92 (2.35e-02)
		wNTI	0.37	-0.56 (1.30e-02)	–	0.86 (2.67e-02)
		Abundance (λ)	0.80	1 (6.20e-08)	–	–
	Insecta	Species Richness	0.57	0.85 (6.94e-05)	–	–
		NRI (λ)	0.28	7.19 (1.05e-02)	-0.68 (8.99e-03)	–
		NTI	0.12	-0.45 (7.34e-02)	–	–
		wNTI	0.18	-0.53 (3.40e-02)	–	–
		Abundance (λ)	0.80	1 (5.73e-08)	–	–
	Diptera	Species Richness (λ)	0.48	0.79 (4.03e-04)	–	–
		NRI	0.32	7.36 (7.41e-03)	-0.7 (5.93e-03)	–
		NTI	0.27	-0.61	–	–

				(1.12e-02)		
		wNTI	0.23	-0.57 (1.98e-02)	–	–
		Abundance (λ)	0.80	1 (7.10e-08)	–	–
Extinction	Invertebrate	Δ Species Richness (λ)	0.45	–	–	-1.35 (6.95e-04)
		Δ NRI	0.34	-0.82 (4.13e-03)	–	–
		Δ wNRI	0.25	-0.72 (1.44e-02)	–	–
		Δ NTI	0.24	-0.71 (1.62e-02)	–	–
		Δ wNTI	0.31	-0.79 (6.37e-03)	–	–
		Δ Abundance(λ)	0.91	-1.2 (2.18e-10)	–	-0.72 (3.56e-05)
	Insecta	Δ Species Richness (λ)	0.48	–	–	-1.39 (4.00e-04)
		Δ Abundance (λ)	0.96	-1.27 (1.03e-13)	–	-0.57 (4.24e-06)
	Diptera	Δ Species Richness (λ)	0.45	–	–	-1.35 (7.59e-04)
		Δ NTI	0.22	-1.01 (2.51e-02)	–	–
		Δ Abundance(λ)	0.96	-1.28 (6.09e-14)	–	-0.5 (1.66e-05)

Table S3. Phylogenetic signal of prey habitat-size preference and predator avoidance during the colonization experiment across different taxonomic scales. Only species with more than one occurrence were included in the analyses. *p*-values for signal statistics are given in parentheses.

Trait	Scale	Blomberg's K	K*	Cmean	Pagel's λ	Moran's I
Size preference	Invertebrate	0.38 (0.55)	0.47 (0.53)	0.08 (0.2)	0.0 (1)	-0.03 (0.47)
	Insecta	0.52 (0.57)	0.55 (0.50)	0.07 (0.22)	0.0 (1)	-0.04 (0.53)
	Diptera	0.55 (0.47)	0.56 (0.47)	0.07 (0.22)	0.11 (0.76)	-0.04 (0.49)
Predator avoidance	Invertebrate	0.70 **(0.01)**	0.68 **(0.02)**	0.21 **(0.02)**	0.51 (0.09)	0.03 **(0.01)**
	Insecta	0.63 (0.22)	0.65 (0.16)	0.00 (0.35)	0.1 (1)	-0.02 (0.14)
	Diptera	0.64 (0.18)	0.65 (0.18)	0.00 (0.39)	0.14 (0.64)	-0.02 (0.16)

References

1. W. P. Maddison, D. R. Maddison, Mesquite: a modular system for evolutionary analysis. (2019). Deposited 2019.

2. A. Stamatakis, RAxML version 8: a tool for phylogenetic analysis and post-analysis of large phylogenies. *Bioinformatics* **30**, 1312–1313 (2014).

3. J. Zhang, P. Kapli, P. Pavlidis, A. Stamatakis, A general species delimitation method with applications to phylogenetic placements. *Bioinformatics* **29**, 2869–2876 (2013).

4. B. Misof, *et al.*, Phylogenomics resolves the timing and pattern of insect evolution. *Science* **346**, 763–767 (2014).

5. M. dos Reis, *et al.*, Uncertainty in the Timing of Origin of Animals and the Limits of Precision in Molecular Timescales. *Current Biology* **25**, 2939–2950 (2015).

6. J. Lozano-Fernandez, *et al.*, A molecular palaeobiological exploration of arthropod terrestrialization. *Phil. Trans. R. Soc. B* **371**, 20150133 (2016).

7. OpenTreeOfLife, *et al.*, Open Tree of Life Synthetic Tree. Deposited 2019.

8. R. R. Bouckaert, *et al.*, BEAST 2.5: An advanced software platform for Bayesian evolutionary analysis. *PLoS Comput Biol* **15**, e1006650 (2019).

9. R. R. Bouckaert, A. J. Drummond, bModelTest: Bayesian phylogenetic site model averaging and model comparison. *BMC Evolutionary Biology* **17**, 42 (2017).

10. A. Rambaut, A. J. Drummond, D. Xie, G. Baele, M. A. Suchard, Posterior summarization in Bayesian phylogenetics using Tracer 1.7. *Systematic biology* **67**, 901–904 (2018).

11. M. Benton, *et al.*, Constraints on the timescale of animal evolutionary history. *Palaeontologia Electronica* (2015). https://doi.org/10.26879/424.

12. H. Chen, *et al.*, A Cambrian crown annelid reconciles phylogenomics and the fossil record. *Nature* **583**, 249–252 (2020).

13. J. M. Wolfe, A. C. Daley, D. A. Legg, G. D. Edgecombe, Fossil calibrations for the arthropod Tree of Life. *Earth-Science Reviews* **160**, 43–110 (2016).

14. C. Cai, *et al.*, Integrated phylogenomics and fossil data illuminate the evolution of beetles. *Royal Society Open Science* **9**, 211771 (2022).

15. E. D. Lukashevich, G. C. Ribeiro, Mesozoic fossils and the phylogeny of Tipulomorpha (Insecta: Diptera). *Journal of Systematic Palaeontology* **17**, 635–652 (2019).

16. J. G. Ogg, C. Huang, L. Hinnov, Triassic timescale status: a brief overview. *Albertiana* **41**, 3–30 (2014).

17. B. M. Wiegmann, *et al.*, Episodic radiations in the fly tree of life. *Proc. Natl. Acad. Sci. U.S.A.* **108**, 5690–5695 (2011).

18. J. Ansorge, Tanyderidae and Psychodidae (Insecta: Diptera) from the Lower Jurassic of northeastern Germany. *Paläont. Z.* **68**, 199–210 (1994).

19. S. P. Hesselbo, J. G. Ogg, M. Ruhl, L. A. Hinnov, C. J. Huang, “Chapter 26 - The Jurassic Period” in *Geologic Time Scale 2020*, F. M. Gradstein, J. G. Ogg, M. D. Schmitz, G. M. Ogg, Eds. (Elsevier, 2020), pp. 955–1021.

20. E. D. Lukashevich, A. A. Przhiboro, F. Marchal-Papier, L. Grauvogel-Stamm, The oldest occurrence of immature Diptera (Insecta), Middle Triassic, France. *Annales de la Société entomologique de France (N.S.)* **46**, 4–22 (2010).

21. P. S. Cranston, N. B. Hardy, G. E. Morse, A dated molecular phylogeny for the Chironomidae (Diptera). *Systematic Entomology* **37**, 172–188 (2012).

22. E. D. Lukashevich, The oldest occurrence of Chaoboridae (Insecta: Diptera). *euroasentj* **31**, 417–421 (2022).

23. J. G. Ogg, Z.-Q. Chen, M. J. Orchard, H. S. Jiang, “Chapter 25 - The Triassic Period” in *Geologic Time Scale 2020*, F. M. Gradstein, J. G. Ogg, M. D. Schmitz, G. M. Ogg, Eds. (Elsevier, 2020), pp. 903–953.

24. G. Poinar, T. J. Zavortink, A. Brown, Priscoculex burmanicus n. gen. et sp.(Diptera: Culicidae: Anophelinae) from mid-Cretaceous Myanmar amber. *Historical Biology* (2019).

25. G. Shi, *et al.*, Age constraint on Burmese amber based on U–Pb dating of zircons. *Cretaceous research* **37**, 155–163 (2012).

26. J. Ansorge, Insekten aus dem oberen Lias von Grimmen (Vorpommern, Norddeutschland). *Neue paläontologische Abhandlungen* **2**, S. 1-132 (1996).

27. W. Krzeminski, E. Krzeminska, Triassic Diptera: descriptions, revisions and phylogenetic relations. *Acta zoologica cracoviensia* **46**, 153–184 (2003).

28. V. Blagoderov, D. A. Grimaldi, N. C. Fraser, How Time Flies for Flies: Diverse Diptera from the Triassic of Virginia and Early Radiation of the Order. *American Museum Novitates* **3572**, 1–39 (2007).

29. T. Sikora, M. Jaschhof, M. Mantič, D. Kaspřák, J. Ševčík, Considerable congruence, enlightening conflict: molecular analysis largely supports morphology-based hypotheses on Cecidomyiidae (Diptera) phylogeny. *Zoological Journal of the Linnean Society* **185**, 98–110 (2019).

30. V. Kovalev, “Diptera” in *Late Mesozoic Insects of Eastern Transbaikalia*, (Nauka Press, 1990), pp. 123–177.

31. D. A. Grimaldi, Diverse Orthorrhaphan Flies (Insecta: Diptera: Brachycera) in Amber From the Cretaceous of Myanmar: Brachycera in Cretaceous Amber, Part VII. *Bulletin of the American Museum of Natural History* **408**, 1–131 (2016).

32. R. Coram, E. A. Jarzembowski, M. B. Mostovski, Two Rare Eremoneuran Flies (Diptera: Empididae and Opetiidae) from the Purbeck Limestone Group. *Paleontological Journal* **34**, S370–S373 (2000).

33. D. A. Grimaldi, Basal Cyclorrhapha in Amber from the Cretaceous and Tertiary(Insecta: Diptera), and Their Relationships: Brachycera in Cretaceous Amber Part IX. *Bulletin of the American Museum of Natural History* **423**, 1–97 (2018).

34. I. S. Winkler, C. C. Labandeira, T. Wappler, P. Wilf, Distinguishing Agromyzidae (Diptera) Leaf Mines in the Fossil Record: New Taxa from the Paleogene of North America and Germany and Their Evolutionary Implications. *J. Paleontol.* **84**, 935–954 (2010).
